# Improving birth weight measurement and recording practices in Kenya and Tanzania: a prospective intervention study with historical controls

**DOI:** 10.1186/s12963-023-00305-x

**Published:** 2023-05-10

**Authors:** Alloys K’Oloo, Evance Godfrey, Annariina M. Koivu, Hellen C. Barsosio, Karim Manji, Veneranda Ndesangia, Fredrick Omiti, Mohamed Bakari Khery, Everlyne D. Ondieki, Simon Kariuki, Feiko O. ter Kuile, R. Matthew Chico, Nigel Klein, Otto Heimonen, Per Ashorn, Ulla Ashorn, Pieta Näsänen-Gilmore

**Affiliations:** 1 Kenya Medical Research Institute, Centre for Global Health Research, P.O. Box 1578-40100, Kisumu, Kenya; 2 Muhimbili National Hospital, Malik/Kalenga Road, P.O. Box 65000, Dar es Salaam, Tanzania; 3 Muhimbili University of Health and Allied Sciences, United Nations Rd, P.O. Box 65001, Dar es Salaam, Tanzania; 4 Faculty of Medicine and Health Technology, Tampere Center for Child, Adolescent and Maternal Health Research, Tampere University, Arvo Ylpön katu 34, 33014 Tampere, Finland; 5 Department of Clinical Sciences, Liverpool School of Tropical Medicine, Pembroke Place, Liverpool, L3 5QA UK; 6 Department of Disease Control, Faculty of Infectious & Tropical Diseases, London School of Hygiene and Tropical Medicine, Keppel St, London, WC1E 7HT UK; 7 UCL Great Ormond Street Institute of Child Health, 30 Guilford Street, London, WC1N 1EH UK; 8 Department of Paediatrics, Tampere University Hospital, PO BOX 2000, 33521 Tampere, Finland; 9 Department for Public Health and Welfare, Public Health Unit, Finnish Institute for Health and Welfare, P.O. Box 30, FI-00271 Helsinki, Finland

**Keywords:** Low birth weight (LBW), Measurement, Accuracy, Digital scales, Data quality, Low- and middle-income countries (LMIC)

## Abstract

**Background:**

Low birth weight (LBW) is a significant public health concern given its association with early-life mortality and other adverse health consequences that can impact the entire life cycle. In many countries, accurate estimates of LBW prevalence are lacking due to inaccuracies in collection and gaps in available data. Our study aimed to determine LBW prevalence among facility-born infants in selected areas of Kenya and Tanzania and to assess whether the introduction of an intervention to improve the accuracy of birth weight measurement would result in a meaningfully different estimate of LBW prevalence than current practice.

**Methods:**

We carried out a historically controlled intervention study in 22 health facilities in Kenya and three health facilities in Tanzania. The intervention included: provision of high-quality digital scales, training of nursing staff on accurate birth weight measurement, recording and scale calibration practices, and quality maintenance support that consisted of enhanced supervision and feedback (prospective arm). The historically controlled data were birth weights from the same facilities recorded in maternity registers for the same calendar months from the previous year measured using routine practices and manual scales. We calculated mean birth weight (95% confidence interval CI), mean difference in LBW prevalence, and respective risk ratio (95% CI) between study arms.

**Results:**

Between October 2019 and February 2020, we prospectively collected birth weights from 8441 newborns in Kenya and 4294 in Tanzania. Historical data were available from 9318 newborns in Kenya and 12,007 in Tanzania. In the prospective sample, the prevalence of LBW was 12.6% (95% confidence intervals [CI]: 10.9%–14.4%) in Kenya and 18.2% (12.2%–24.2%) in Tanzania. In the historical sample, the corresponding prevalence estimates were 7.8% (6.5%–9.2%) and 10.0% (8.6%–11.4%). Compared to the retrospective sample, the LBW prevalence in the prospective sample was 4.8% points (3.2%–6.4%) higher in Kenya and 8.2% points (2.3%–14.0%) higher in Tanzania, corresponding to a risk ratio of 1.61 (1.38–1.88) in Kenya and 1.81 (1.30–2.52) in Tanzania.

**Conclusion:**

Routine birth weight records underestimate the risk of LBW among facility-born infants in Kenya and Tanzania. The quality of birth weight data can be improved by a simple intervention consisting of provision of digital scales and supportive training.

## Background

Low birth weight (LBW), defined by the World Health Organization (WHO) as a birth weight less than 2500 g [1], is a major contributor to neonatal mortality [2–4]. The LBW infants who survive infancy are at increased risk of long-term sequelae, including neurodevelopmental problems [5]; stunting [6]; respiratory disorders [7]; lower IQ [8]; and adult-onset chronic diseases [9–12]. Additionally, when LBW girls become mothers, they are more likely to deliver LBW infants themselves [13]. Thus, reducing the incidence of LBW has public health implications that are lifelong and inter-generational.

Globally, approximately 15% of live births are LBW, with the highest burden in southern Asia and Sub-Saharan Africa [14]. However, the true magnitude is likely underestimated due to poor data coverage and quality. Poor data coverage may result from home births where newborn weight is rarely measured or recorded [15–17] or when the neonate is frail, ill, or dies [15]. Alternatively, newborns may be weighed, but the measurement is not recorded in routine data sources such as health cards [18]. Birth weight data may also be inaccurately recorded for various reasons. A common preference for the terminal digit 0 or 5 has been described [19, 20] and heaping of birth weight data, which involves rounding birth weights to the closest 100 g or 500 g interval. For example, infants weighing 2,490 g are recorded as 2,500 g. This practice leads to inaccurate estimates of LBW [21, 22] at the individual and population levels [15, 20, 23]. Other barriers to accurate birth weight data are random and systematic measurement errors [24], including delays in birth weight measurement until several days after birth [24, 25], or subtracting the estimated weight of clothes after measuring a dressed or swaddled newborn [21]; inaccurate, unavailable or inaccessible scales [21, 24, 26]; lack of standardized technical weighing protocols [21]; or poor calibration of scales [14]. Complicated register design may further contribute to inaccuracies in birth weight data [27]. Finally, various health system or sociocultural factors, including limited understanding of why the data are collected, may underpin sub-optimal data quality and use [28].

Accurate birth weight measurement serves as a guide to appropriate care for newborns. Also measuring birth weight provides a low-cost, feasible method of monitoring newborn health which is globally applicable. It is also vitally important metric in monitoring neonatal outcomes at the population level and tracking national, regional, and global progress towards the Every Newborn Action Plan [29], the Global Nutrition Plan [1], and Sustainable Development Goals [30]. These global initiatives may be particularly important for LMICs, the same countries that face the most barriers to reliable LBW data. Against this background, we estimated the prevalence of LBW or all birth weights less than 2500 g among infants born at selected time periods in two LMICs and evaluated a simple intervention designed to improve LBW measurements with the aim of answering two research questions:

1. What is the prevalence of LBW among infants born in health facilities in selected health areas in Kenya and Tanzania?
2. Will the introduction of a support package (use of improved scales, training and enhanced staff supervision and feedback) result in different LBW prevalence estimate compared to current practices in the same health facilities?

By addressing these questions, we sought to contribute to the improved understanding of existing newborn weighing methods in low-resource settings and improve the accuracy of measurements with simple intervention.

## Methods

### Study design

**Fig. 1:**
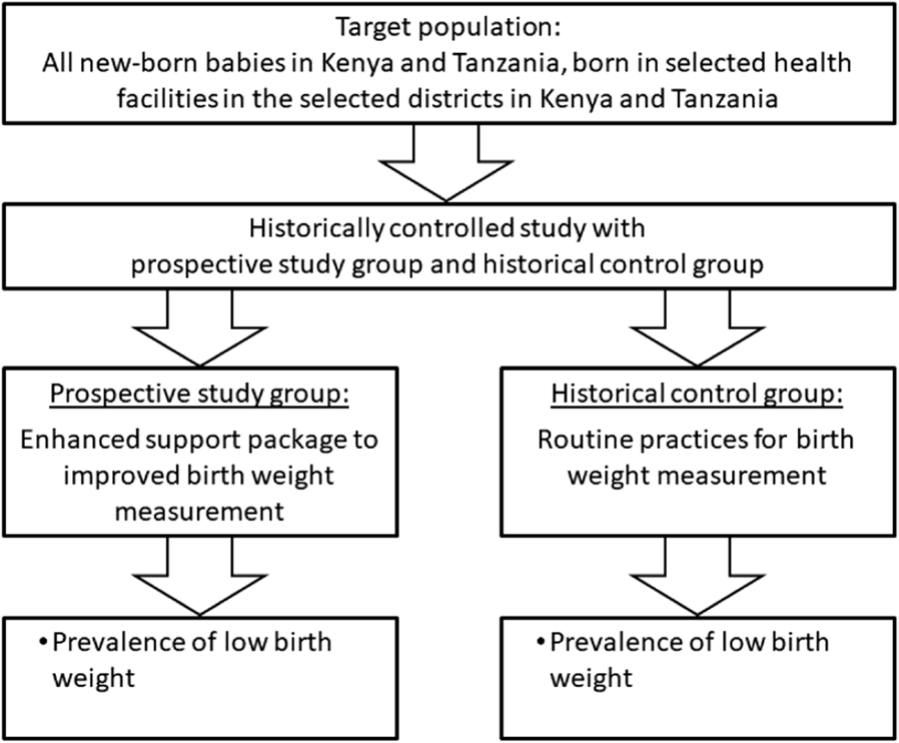
Study design: a prospective intervention study with historical controls

We conducted a prospective study with historical controls at 22 health facilities in Kenya and three health facilities in Tanzania to improve birth weight measurement and recording practices (Fig. 1). We focused on birth weight data only. We did not collect data on gestational age as these data were not available due to a lack of ultrasound monitoring as part of antenatal care routines at study facilities. The prospective component was conducted for 4.5 months from October 2019 to February 2020, targeting all neonates born during the study period (prospective arm). Historical birth weight data consisted of previously recorded birth weight data for all births from the same selected health facilities during the same calendar months of the preceding year (historical arm).

### Study context

Kenya and Tanzania are representative of LMICs where access to accurate and timely LBW data is a challenge [17, 21, 31]. In Kenya, the latest country-level LBW estimate, 11.5% (95% CI: 8.9–14.5) of live births, is from 2015 [18]. The Kenya Demographic and Health Survey 2014 estimated LBW prevalence to be 7.6% among newborns with a reported birth weight [32]. In Tanzania, the country prevalence of LBW was estimated at 10.5% (95% CI: 8.1–13.4) in 2015 [18]. Additionally, Tanzanian record-based studies have previously reported prevalence rates of 9.5% to 20.5% between 2000–2010 and 2010–2015, respectively, depending on the study setting and population [33, 34], suggesting considerable in-country variation.

### Study sites

The study sites were selected to represent the whole range of delivery facilities in urban and rural settings capturing variation in numbers of births per year, infrastructure, average county-level birth rate, and staffing and supervision. We selected study facilities if their maternity registers contained complete birth information, including birth date, birth weight, and sex of the newborns for the year preceding the study period. In Kenya, the study was conducted in 22 rural health facilities in four counties (Homa Bay, Siaya, Migori, and Kisumu). In Tanzania, the study was conducted in two referral hospitals (Amana and Temeke) and one health centre (Mbagala Rangitatu) in suburban Dar es Salaam (Table 1). In Kenya, the study facilities represented a majority of the facilities in the area. In Tanzania, the facilities in received highest numbers of antenatal cases in the area and catered for almost 60–70% of the population of Dar es Salaam.

**Table 1:** Data collection sites in study countries chosen to represent the whole range of variation in the source population

Country	County / site	No. of health facilities included	Type of health facilities by level^1^	No. of deliveries per month
Kenya	Kisumu County	8	3, 4, 5	680
	Siaya County	5	3, 4, 5	410
	Homa Bay County	5	3, 4, 5	420
	Migori County	4	3, 4, 5	340
Tanzania	Temeke Regional Referral Hospital	1	4	1000
	Mbagala Rangi	1	3	1000
	Amana Regional Referral Hospital	1	4	900

^1^The six levels of health care service delivery are 1 community, 2 dispensaries, 3 health centre,

4 district hospital, 5 provincial hospitals, 6 national referral hospital [34, 35]

### Intervention

The support package provided to the prospective group consisted of:

1. Provision of digital weighing scales with 10 g reading increment.
2. Training of health workers in scale calibration and use and in precise birth weight recording.
3. Monitoring and evaluation: Quality maintenance support via setting up a weekly supervision and feedback routine for senior nurses and facility managers on the intervention uptake and data quality.
4. Scheduled mid-point retraining to reinforce the effectiveness of the intervention.

The intervention was fitted into the existing newborn weighing and care routines of the selected health facilities as much as possible.

*Provision of scales* Before the intervention, the facilities used traditional analogue weighing scales or hand-held scales. We provided each study facility a battery-operated digital scale (Seca 354, Seca, Hamburg, Germany) that measures in graduations of 10 g. Larger facilities with separate operating theatres were given two scales. We also provided standard weights (0.5 kg, 1.0 kg, 2 kg, 3 kg) for calibration. In some facilities, official calibration stones of similar weight were already available. If these existing calibration stones were used, we ensured that they produced correct readings and were in good condition with no visible chippings, affecting the weight.

*Training* Prior to data collection, we carried out a three-day training among staff members from labour, post-natal, and natal wards and obstetric theatres of the selected health facilities. For the larger facilities in Kenya, we trained health facility supervisors who then trained nurses, whereas in smaller facilities in Kenya and all facilities in Tanzania, we trained nurses. Participants were provided with a detailed outline of the study, highlighting the importance of accurate birth weight measurement. Nurses were trained to weigh naked newborns within one hour of birth, using the digital scale provided on a level hard surface and recording the result with 10 g precision. A standard operating procedure was provided to ensure uniformity of performance. We also trained the nurses to calibrate the digital scales and provided a daily calibration sheet in which the nurses were asked to record the status of calibration at the start of each working day. Scale readings within the limit of ± 20 g were considered acceptable, and any readings beyond the accepted range were reported. In addition, nurses were asked to record all birth data into the maternity register following the existing routine practices. We tested whether the amount of training was adequate at each health facility for a period of two days following the training. We randomly selected nurses from each facility who practised daily calibration of the scales, invited mothers for routine newborn weighing, and practised the improved birth weight measuring techniques using the digital scales.

*Monitoring and Evaluation* As a method of monitoring and evaluation, we designed our own system to measure the efficacy of the intervention implementation and quality control. Health facility supervisors and district nurses were asked to reinforce the training of health workers and midwives through enhanced supervision and feedback on-site. They were also instructed to report to the research team on the quality of work using a daily calibration log and weekly supervision logs. Furthermore, they were asked to ensure with regularity the accuracy and completeness of the data by weekly reporting of training and supervision frequency received at study sites, numbers of correct scales calibrations completed, numbers of days when birth records were correctly completed, proportion of rounding of birth weight, and organizing mid-point interviews to obtain feedback on the process from health workers. In Kenya, depending on the site, health facility supervisors or district nurses visited the sites once a week supported and supervised by the central research team. In Tanzania, monitoring of quality maintenance was conducted by a member of the research team who visited the sites weekly.

*Retraining* At the mid-point of the data collection, the central research team in each country visited the study locations to reinforce the effect of the support package by running a second training session at all sites. The training consisted of the same topics as the first session but was reduced to half a day. At smaller sites, the research team trained the nurses and midwives, whereas, at the larger sites, the research team trained the district nurses, i.e. the trainers of the nurses and midwives.

### Data collection and entry

We collected data from maternity records for the prospective and historically controlled groups. We obtained data on maternity ID, birth weight in grams, sex of the newborn (male/female/not recorded), and date of birth (dd, mm, yyyy). In Kenya, for prospective data, field supervisors collected data weekly and entered data into a tablet with CommCare data collection software (Dimagi Inc., USA). For historical data, the field supervisors photographed the historically controlled data and entered them into the CommCare application. In Tanzania, both historical and prospective data were photographed and entered into an Excel spreadsheet (Microsoft Excel, USA). In both countries, collected variables were the same, and the data were entered in the dataset by two people independently. The two datasets were linked using maternity ID and cross-checked by data managers in the research team. If any discrepancies were identified, the data were checked from the original health records.

### Statistical analyses

We calculated mean birth weight and 95% confidence interval (CI). We also compared the mean birth weight between the prospective intervention group and the historical control groups using the Student’s independent *t*-test (Stata 16, Stata Corp., USA). The outcome variable (birth weight) was grouped into LBW (< 2500 g) and normal birth weight (≥ 2500 g) in the categorical analysis. Although rounding and digit preference occur throughout the birth weight range, we focused our analyses at the 2500 g cut-off. This is an internationally recognized cut-off for LBW [1], and inaccuracy in measuring it is likely to have direct effects local, national, regional, and global health statistics as well as individual newborn management and programmes [15, 20, 23]. We calculated the absolute difference and risk ratio (and 95% CIs) in LBW prevalence between the prospective and historical arms. The analyses were adjusted for the cluster (health facility). Additionally, due to the known practice of digit preference resulting in heaping of birth weight data on multiples of 500 g. We calculated an adjusted LBW prevalence by reallocating 25% of infants with an exact birth weight of 2500 g to the LBW category. This adjustment method has been used in previous studies [23, 27].

## Results

**Fig. 2:**
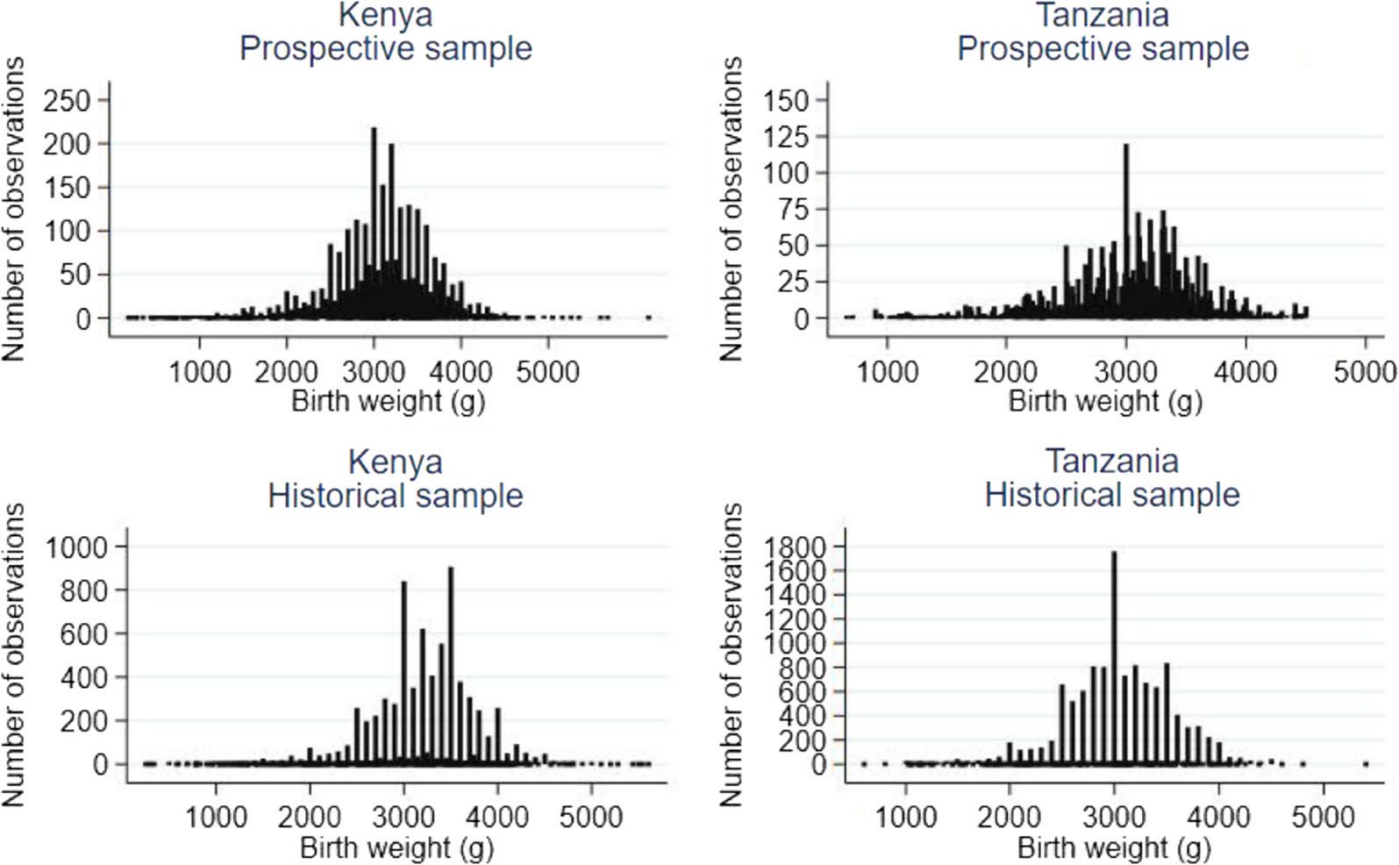
Birth weight distribution in Kenya and Tanzania: prospective and historical sample

Between October 2019 and February 2020, we prospectively collected birth weights from 8441 newborns in Kenya and 4294 in Tanzania. Historical data were available from 9318 newborns in Kenya and 12,007 in Tanzania (Table 2). The prospectively recorded birth weights ranged from 600 to 5890 g in Kenya and 700 to 4600 g in Tanzania. The birth weights based on historical data ranged from 650 to 5700 g in Kenya and 690 to 5500 g in Tanzania (Fig. 2).

**Table 2:** Prevalence of birth weights below 2,500 g (LBW) in Kenya and Tanzania in prospective and historical sample

	Prospective Sample (95% CI)	Historical Sample (95% CI)	Absolute difference in LBW prevalence (95% CI)	Risk ratio (95% CI)
*Kenya *				
Number of births	8441	9318	N/A	N/A
Proportion of birth weights < 2500 g	12.6% (10.9; 14.4)	7.8% (6.5; 9.2)	4.8% (3.2; 6.4)	1.61 (1.38; 1.88)
Adjusted LBW% proportion of birth weights < 2500 g*	12.9% (11.2; 14.6)	8.5% (7.1; 9.9)	4.3% (2.7; 6.0)	1.51 (1.29; 1.77)
*Tanzania *				
Number of observations	4294	12,007	N/A	N/A
Proportion of birth weights < 2500 g	18.2% (12.2; 24.2)	10.0% (8.6; 11.4)	8.2% (2.3; 14.0)	1.81 (1.30; 2.52)
Adjusted LBW% proportion of birth weights < 2500 g*	18.5% (12.7; 24.2)	11.4% (9.9; 12.9)	7.1 (1.9; 12.3)	1.62 (1.23; 2.13)

*Adjusted LBW prevalence was calculated after reallocating 25% of 2500 g infants to be LBW

The mean (SD) birth weight in Kenya was 3080 g (590) in the prospective sample and 3190 g (570) in the historical sample. In Tanzania, the respective values were 2990 g (590) and 3030 g (530). Compared to the historical sample, the mean birth weight in the prospective sample was 110 g (95% CI: 100–130) lower in Kenya and 40 g (95% CI: 20–60) lower in Tanzania.

In Kenya, the prevalence of LBW assessed prospectively (*N* = 8,441) was 12.6% (95% CI: 10.9%–14.4%) compared to 7.8% (6.5%–9.2%) in the historical data (*N* = 9318) (absolute difference 4.8% points (95% CI: 3.2%–6.4%), risk ratio 1.61 (1.38–1.88)). The corresponding figures in Tanzania were 18.2% (95% CI: 12.2%–24.2%) prospectively (*N* = 4294) and 10.0% (95% CI: 8.6%–11.4%) retrospectively (*N* = 12,007) (absolute difference 8.2% points (95% CI: 2.3%–14.0%), risk ratio 1.81 (1.30–2.52)). A sensitivity analysis using adjusted birth weights by reallocating 25% of infants with an exact birth weight of 2,500 g to the LBW category gave essentially similar results in both countries (Table 2).

## Discussion

The purpose of this study was to determine a prevalence of LBW among infants born in selected health facilities in two LMIC countries and to assess whether a simple intervention to improve the accuracy of birth weight measurement would produce a different estimate of LBW prevalence than the current practice. The provision of new digital scales, training of nurses, and quality maintenance support resulted in a higher estimate of LBW prevalence: 4.8 percentage points (from 7.8 to 12.6%) in Kenya and 8.2 percentage points (from 10.0 to 18.2%) in Tanzania compared to historical sample produced using routine practices in a sample of approximately 34,000 facility births.

The validity of the findings could theoretically have been affected by variation in birth weight measurement in the multiple study facilities, but we minimized this problem by enhanced supervision, communication and clear accountability structures. Moreover, the study facilities might be considered “high performers” given that they contained complete birth information from the previous year. The differences between prospective and historical data might have been greater in facilities with incomplete birth information. In Tanzania, the number of births was lower in the prospective sample than the historical one because a new birthing facility was opened in the study area during the period of prospective data collection. This might theoretically have led to the referral of low-risk mothers to this new facility and an increase in the proportion of high-risk deliveries in the study facilities and thus potentially have contributed to an increase in the proportion of LBW infants in our prospective sample. There was, however, no public recommendation to concentrate or refer certain types of deliveries to specific facilities. Because of this and the consistency of the findings, we believe that our findings are valid and indicate that routine birth weight recording has produced considerable underestimates in the prevalence of LBW.

Observer errors have been reported in birth weight research [36]. Weights ending in multiples of 100 or 500 g tend to be preferred, resulting in a heaping of birth weight measurements [15, 19, 20]. This affects LBW estimates, particularly in LMICs [22]. While heaping is less common in birth weight data from health cards than from maternal recall, the practice can still significantly affect LBW estimates. For example, in a comparison of six LMICs, 10% to 64% of birth weights on health cards were recorded in multiples of 500 g or ½ kg [22]. To address this, we frequently highlighted in training the importance of precision and introduced digital scales, which reduces measurement heaping [27, 37]. While there was still some heaping, even in our prospective sample, it did not significantly affect the estimate of the LBW prevalence or the impact of the study intervention on it, as evidenced by the sensitivity analysis. Furthermore, it is clear that a simple intervention can significantly improve data accuracy and enhance the identification of LBW babies and, at the individual level, contribute to better targeting of newborns who are most in need of care. At the population level, this will contribute to more accurate resource allocation for small and vulnerable newborns.

The 12.4% LBW prevalence in Kenya and 18.2% in Tanzania are higher than the UNICEF reported national prevalence rates from 2015 (11.5% for Kenya and 10.5% for Tanzania [18]) and higher than the estimates in Demographic Health Surveys for the study locations [32, 38], but roughly in line with the regional rate for sub-Saharan Africa which was estimated to be 12.2–17.2% in 2015 [14]. Demographic Health Surveys are likely to underestimate the prevalence of LBW due in part to accuracy issues previously discussed, reliance in some cases on maternal recall, and the notable proportion of newborns who are not weighed [15].

We did not identify other controlled studies to achieve more precise LBW estimates through an intervention targeted at the facility level. An Indian hospital-based study weighed 859 live births using analogue and digital scales and found that significantly more newborns weighed exactly 2500 g on analogue versus digital scales. The prevalence of LBW by digital scale (29.5%) was significantly higher compared to the analogue device (23.0%) [37]. Data quality interventions using various forms of support, including training, reviews, audits and feedback, have improved accuracy, completeness, timeliness, and other quality aspects in maternal and newborn health data in LMICs [39–41]. However, these types of studies do not typically provide before and after LBW estimates. The EN-BIRTH study examined labour and delivery ward register data availability, quality, and utility and identified significant heaping of birth weights in Bangladesh, Nepal, and Tanzania [42]. However, the study design included no intervention to address the deficiencies in LBW data quality. The study also compared the birth weight data in hospital registers and women’s report at exit interview survey [27] and found that the register-based LBW rate was 14.9% for the three countries, and the rate was slightly higher and more specific and sensitive than survey-based rate. The qualitative component of the EN-BIRTH study explored barriers and enablers to weighing birth in Temeke Hospital, which is one of the sites of the current study. Our intervention directly addressed many of the gaps they reported, including the lack of precise equipment and standardized technical weighing protocols. A recent study from Ethiopia reports the effect of data quality intervention on LBW prevalence before and after the intervention and provides interesting qualitative insights into the success of the intervention [43].

The LBW prevalences reported in the current study are not generalizable beyond the study areas and cannot be taken as indicators of national LBW prevalence in Kenya and Tanzania. This is because data collection took place only at health facilities and included no data from home deliveries that remain common in both countries. Furthermore, the study samples were not designed to be nationally representative, as they represented only limited numbers of geographic areas and only some types of maternity wards. However, while these issues obviously limit the generalizability of the reported LBW prevalences, they do not affect the main conclusions of the study.

## Conclusions

There has been a call for research to establish the efficacy and feasibility of interventions to improve the quality of birth weight data [20]. Our study demonstrated that routine birth weight data markedly underestimates LBW prevalence. Furthermore, it showed that a simple intervention introducing only modest changes in existing daily practices at health facilities can lead to significantly more accurate birth weight data in low-resource settings and thus contribute to more precise LBW estimates. This relatively low-cost intervention, which does not require excessive training, is readily deliverable in LMICs beyond the study countries and has the potential to improve birth weighing and data recording practices with great potential to improve newborn survival globally.

## Data Availability

The datasets used and/or analysed during the current study are available from the corresponding author on reasonable request.

## Abbreviations

BW: Birth weight
CI: Confidence interval
IRB: Institution Review Board
KEMRI: Kenya Medical Research Institute
LBW: Low birth weight
LMIC: Low- and middle-income country
MUHAS: Muhimbili University of Health and Allied Sciences
MNH: Muhimbili National Hospital
MoH: Ministry of Health
NIMR: National Institute of Medical Research
WHO: World Health Organization
